# Relationship between alcohol-attributable disease and socioeconomic status, and the role of alcohol consumption in this relationship: a systematic review and meta-analysis

**DOI:** 10.1186/s12889-015-1720-7

**Published:** 2015-04-18

**Authors:** Lisa Jones, Geoff Bates, Ellie McCoy, Mark A Bellis

**Affiliations:** Centre for Public Health, Faculty of Education, Health and Community, Liverpool John Moores University, Henry Cotton Campus, Level 2, 15-21 Webster Street, Liverpool, L3 2ET UK; Policy, Research and Development, Public Health Wales, Haydn Ellis Building, Maindy Road, Cardiff, CF24 4HQ UK

**Keywords:** Alcohol, Socioeconomic status, Morbidity, Mortality, Systematic review

## Abstract

**Background:**

Studies show that alcohol consumption appears to have a disproportionate impact on people of low socioeconomic status. Further exploration of the relationship between alcohol consumption, socioeconomic status and the development of chronic alcohol-attributable diseases is therefore important to inform the development of effective public health programmes.

**Methods:**

We used systematic review methodology to identify published studies of the association between socioeconomic factors and mortality and morbidity for alcohol-attributable conditions. To attempt to quantify differences in the impact of alcohol consumption for each condition, stratified by SES, we (i) investigated the relationship between SES and risk of mortality or morbidity for each alcohol-attributable condition, and (ii) where, feasible explored alcohol consumption as a mediating or interacting variable in this relationship.

**Results:**

We identified differing relationships between a range of alcohol-attributable conditions and socioeconomic indicators. Pooled analyses showed that low, relative to high socioeconomic status, was associated with an increased risk of head and neck cancer and stroke, and in individual studies, with hypertension and liver disease. Conversely, risk of female breast cancer tended to be associated with higher socioeconomic status. These findings were attenuated but held when adjusted for a number of known risk factors and other potential confounding factors. A key finding was the lack of studies that have explored the interaction between alcohol-attributable disease, socioeconomic status and alcohol use.

**Conclusions:**

Despite some limitations to our review, we have described relationships between socioeconomic status and a range of alcohol-attributable conditions, and explored the mediating and interacting effects of alcohol consumption where feasible. However, further research is needed to better characterise the relationship between socioeconomic status alcohol consumption and alcohol-attributable disease risk so as to gain a greater understanding of the mechanisms and pathways that influence the differential risk in harm between people of low and high socioeconomic status.

**Electronic supplementary material:**

The online version of this article (doi:10.1186/s12889-015-1720-7) contains supplementary material, which is available to authorized users.

## Background

Alcohol consumption is common in industrialised countries and globally represents the fifth largest single cause of premature mortality, loss of health and disability [1]. In 2010, alcohol use resulted in 2.7 million deaths and accounted for around 4% of global disability-adjusted life years [1]. Studies reveal a complex association between alcohol consumption and socioeconomic status (SES). While many studies have found that the burden of alcohol-related mortality and morbidity falls most heavily on people of low SES [2-7], actual alcohol consumption patterns tend to show a deviation from the traditional pattern observed with risky health behaviours. This gives rise to a paradox whereby disadvantaged populations that apparently have the same, or a lower level, of alcohol consumption suffer greater alcohol-related harm than more affluent populations.

How alcohol consumption affects the risks of health conditions has been well characterised. At lower levels of consumption, studies suggest alcohol consumption is associated with both increased health risks for some conditions (e.g. cancers, liver cirrhosis) and decreased for others (e.g. ischaemic heart disease, ischaemic stroke). It also clear that patterns of drinking, as well as volume, play an important role in both the disease burden and health benefits associated with drinking [8]. Further exploration of the relationship between alcohol consumption, SES and the development of alcohol-attributable diseases, however, is important in order to understand the contribution they make to the disproportionate impact that alcohol consumption appears to have on those of low SES and to inform the development of effective public health programmes.

We therefore used systematic review methodology to identify published studies that examined the association between socioeconomic factors and mortality and morbidity for a range of alcohol-attributable conditions. Our primary objective was to attempt to quantify the impact of alcohol consumption for each condition, stratified by SES. Our steps to achieve this objective included using meta-analysis to quantify differences in the risk of alcohol-attributable disease between high and low SES groups for studies with and without adjustment for alcohol use alone or in combination with other behaviours, and exploration of alcohol use as a mediating or interacting variable in this relationship. To our knowledge, no systematic review of this type has been undertaken previously.

## Methods

The methods were based on guidelines for undertaking systematic reviews of observational studies by following the Preferred Reporting Items for Systematic reviews and Meta-analysis statement [9].

### Search strategy

**Figure 1:**
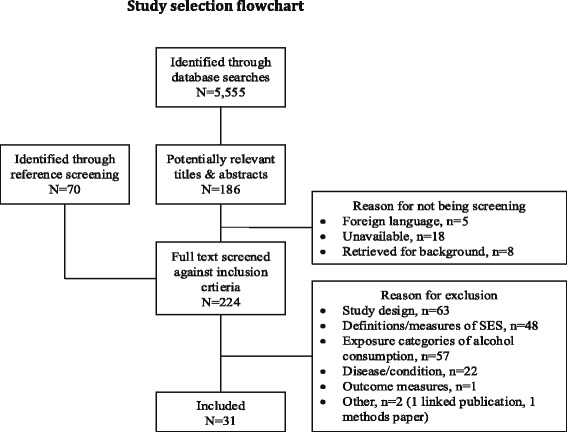
Study selection flowchart.

Searches were undertaken in Medline, Embase, PsycINFO, CINAHL, and the Web of Science in November 2012. A search strategy was developed using a combination of free text and controlled vocabulary terms and adapted for each database. See Additional file 1 for an example search strategy. As a single search strategy was used for a series of reviews on the topic of alcohol-related harm and SES, in addition to terms for diseases partially attributable to alcohol use, the search incorporated terms for wholly attributable conditions and for injuries. References were additionally identified through searches of reference lists. The process of study selection is summarised in Figure 1.

Titles and abstracts identified through the searches were reviewed independently by two reviewers. At this stage we sought to identify studies of any alcohol-attributable condition that reported outcomes according to differing levels of SES (any measure of SES was accepted at this stage). Studies identified as potentially relevant by either reviewer were retrieved for further inspection. Full text copies of the selected studies were retrieved and independently reviewed against the full inclusion criteria by two reviewers from a team of three. Studies were retained if they met the following criteria: (i) case–control or cohort study; (ii) participants were aged 16 years or older; (iii) reported definitions and measurement of SES (including income, occupation, level of education or aggregate measures of neighbourhood-level deprivation); (iv) reported risk, odds or hazard ratios across different exposure categories of alcohol consumption; (v) reported mortality or morbidity outcomes for diseases with a known adverse risk relationship with alcohol consumption (specifically liver disease; hypertension; cancers of the mouth, head and neck, female breast, oesophagus [squamous cell carcinoma], colorectum, and liver; stroke and other cerebrovascular disorders; epilepsy; cardiac arrhythmia; and pancreatitis); (vi) published in the English language.

### Data extraction and quality assessment

Methodological details recorded from studies included study details; participant details; response rates (at baseline/follow-up); follow-up duration; SES measures; and alcohol consumption measures. We also planned to extract (adjusted and unadjusted) risk estimates for each alcohol exposure category, stratified by SES. However this data was not commonly available so we extracted (adjusted and unadjusted) RRs and corresponding 95% confidence intervals (CIs) for each alcohol exposure category and each SES category independently. For measures of SES, the adjusted risk estimates of interest were those that the study authors had attempted to control for alcohol consumption, either alone or in combination with other behavioural risk factors. The measures of SES in the included studies were reported across multiple strata. To simplify comparison across studies we retained the risk estimates comparing only the lowest and highest SES categories, using high SES as the reference category. For studies that reported low SES as the reference category, the reciprocal of the risk estimate was used to recalculate the point estimate and 95% CIs. A small number of studies reported regression coefficients and these were extracted directly but not used in the pooled analyses. Quality was assessed using the Newcastle-Ottawa Scale. Data extraction and quality assessment were undertaken by one reviewer from a team of two and checked for accuracy by a second reviewer from a team of three.

### Statistical analysis

Our planned methods included meta-analysis to generate pooled estimates of the change in risk of alcohol consumption among participants with differing SES. In practice, we identified very few studies that reported alcohol exposure categories stratified by SES. As an alternative method, we used the extracted risk estimates (OR and 95% CI) to quantify differences in the risk of alcohol-attributable disease between high and low SES groups for studies with and without adjustment for alcohol use alone or in combination with other behaviours. As studies provided data on multiple measures of SES, to simplify the analyses and maximise data availability, we used an approach suggested by Lorant et al. [10]. Level of education was retained as the measure of SES when data on multiple measures of SES were reported, and when not available, income was considered next, followed by social occupational class and then neighbourhood level measures. We derived the log OR and corresponding standard error (SE) for each study and they were used in the pooled analyses. All meta-analyses were conducted in Review Manager (version 5.3) using the generic inverse variance outcome type. All analyses were conducted using the random effects model of DerSimonian and Laird method [11]. The *I*^2^ statistic was used to estimate heterogeneity. We planned to assess risk of bias, specifically publication bias, through visual inspection of funnel plots. However, although funnel plots were generated, insufficient studies were included in the meta-analyses to reliably identify sources of asymmetry [12]. Forest plots were generated showing unadjusted and adjusted ORs with corresponding 95% CIs for each study and the overall random-effects pooled estimate. Potential sources of heterogeneity were further investigated by use of visual inspection of the data, forest plots, and funnel plots.

For studies where models were adjusted for alcohol use alone we additionally calculated the percentage change in odds ratio between the highest and lowest SES category brought about by the addition of alcohol use to the unadjusted model. As a third step, we explored the relationship between alcohol use and SES where further analyses of the mediation or interaction between the two measures were presented.

## Results

### Study identification

The search strategy retrieved 5,555 studies and of these, 186 were selected by at least one reviewer as potentially relevant and selected for full text review. Seventy additional studies were identified through reference screening. In total, 31 papers were not screened against the full inclusion criteria; 18 papers were unavailable, 5 papers were foreign language publications and 8 papers were retrieved for background information only. A total of 224 papers were screened against the inclusion criteria and 31 studies selected for inclusion. Reasons for exclusion are summarised in the flowchart in Figure 1.

### Study characteristics

Study characteristics are summarised in Table 1. Eighteen studies examined various types of cancers, six studies [13-18] examined cancers of the head and neck, three studies [19-21] examined oesophageal cancer, eight studies [22-29] examined female breast cancer, two studies [22,28] examined all types of cancer, one study [28] examined smoking and alcohol-related cancers as a group, and one study [30] examined liver cancer. Seven studies [31-37] examined death or hospitalisation from stroke, two studies [38,39] systolic blood pressure (BP), and two further studies risk of hypertension [40,41]. Two additional studies examined atrial fibrillation [42] and liver disease [43]. Seven studies were conducted in the USA [15,16,19,20,29,38,40], four in France [17,28,39,41], four in the UK [14,21,23,35], three in Finland [32,34,36], two in Poland [18,25], two in Italy [37,42] and one each in New Zealand [31], Sweden [33], Norway [22], Denmark [26], Brazil [13], Germany [24], Spain [27], South Korea [30], and Hungary [43]. Seventeen studies [13-21,24,25,27,29,31,41-43] were based on a case–control design and 14 were cohort studies [22,23,26,28,30,32-40]. Quality scores ranged from 5 to 8 out of a possible 9. Study quality assessment is summarised in Table 2 for cohort studies and Table 3 for case-control studies. A range of SES measures were examined across the included studies including level of education [13,15-18,20,22-26,29,31,33,34,36-43], occupational social class [13,16,17,19,21,26-28,30,32,35,37], income [19,20,23,25,26,31,38,42], employment status [15,16,39], home ownership or tenure [38,39], community or area-level SES [14,29] and occupational mobility [28]. Fifteen studies [13,15-20,23,25,26,28,29,38,39,42] reported outcomes for more than one measure of SES. Measures of alcohol use varied across the included studies: 10 studies [15-17,19,20,25,28,29,31,34,41] reported number of drinks or glasses consumed in a day or week; 11 studies [13,22,24,26,27,30,32,33,37,40,42] reported grams or millilitres of alcohol consumed in a day, week or year; four studies [14,21,35,43] reported number of units drank in a week; and one study each, reported a composite measure of number of drinks and days drank in a week [38], a dichotomous measure of drinking in the last 12 months [23]; years of vodka consumption [18]; binge drinking [36], or provided no definition [39].

**Table 1:** **Characteristics of the included studies**

**Author, Year, Country**	**Study years**	**Country**	**Disease area**	**Study type**	**QA score**	**Sex**	**Age, yrs (mean or range)**	**Cases**	**Controls**	**SES measure(s) (no. of strata)**	**Alcohol measure (no. of strata)**
Boing et al., 2011 [13]	1998-2006	Brazil	Head & neck cancer	CC	5	M&F	NR	1,017	951	E (3), O (2)	g/yr (5)
Braaten et al., 2005 [22]	1991-1997	Norway	Cancers [Various]	CO	7	F	30-69	93,638	NA	E (4)	g/d (4)
Brown et al., 2001 [19]	1986-1989	USA	Oesophageal cancer	CC	7	M	30-79	347	1,354	E (3), O (3), I (3)	Drinks/wk (4)
Brown et al., 2005 [31]	1991-1994 & 2003	New Zealand	Stroke	CC	6	M&F	69 cases; 60 controls	1,242	2,247	I (2)	Drinks/d (2)
Brummett et al., 2011 [38]	NR	USA	Systolic BP	CO	6	M&F	29	14,299	NA	E (5), I (13), T (2)	Days drank/wk; Drinks/wk (4)
Chaix et al., 2010 [39]	2007-2008	France	Systolic BP	CO	6	M&F	30-79	5,941	NA	E (4), ES (3), T (2)	Drinking status (4)
Conway et al., 2010 [14]	2002-2004	UK	Head & neck cancer	CC	7	M&F	NR	103	91	A (5)	Units/wk (5)
Day et al., 1993 [15]	1984-1985	USA	Head & neck cancer	CC	5	M&F	18-79	1,065	1,182	E (3), ES (2)	Drinks/wk (5)
Dyer et al., 1999 [40]	1985-1986	USA	Hypertension	CO	8	M&F	18-30	4,747	NA	E (2)	ml/d (NA)
Gammon et al., 1997 [20]	1993-1995	USA	Oesophageal cancer	CC	6	M&F	66	221	695	E (6), I (5)	Drinks/wk (6)
Greenberg et al., 1991 [16]	1984-1985	USA	Head & neck cancer	CC	5	M	18-79	762	837	E (3), O (3), ES (3)	Drinks/wk (5)
Heck & Pamuk, 1997 [23]	1971-1975	UK	Breast cancer	CO	8	F	NR	6,261	NA	E (4), I (5)	Last 12 months (2)
Joshi et al., 2008 [30]	NR	South Korea	Liver cancer	CO	7	M	30-59	548,530	NA	O (4)	g/d (5)
Kivimaki et al., 2009 [32]	2000-2004	Finland	Stroke	CO	7	F	44	48,361	NA	O (3)	g/wk (3)
Kropp et al., 2001 [24]	1992-1995	Germany	Female breast cancer	CC	7	F	43	706	1,381	E (3)	g/d (6)
Kruk et al., 2007 [25]	2003-2007	Poland	Female breast cancer	CC	7	F	55	858	1,085	E (4), I (3)	Drinks/wk (3)
Kuper et al., 2007 [33]	1991-1992	Sweden	Stroke	CO	8	F	40	47,942	NA	E (4)	g/d (4)
Laaksonen, et al., 2008 [34]	1979-2001	Finland	Stroke	CO	7	M&F	25-64	60,608	NA	E (3)	Drinks/wk (3)
Larsen et al., 2011 [26]	1993-1997	Denmark	Female breast cancer	CO	7	F	50-64	23,111	NA	E (3), O (7), I (4)	g/d (NA)
Martin-Moreno et al., 1993 [27]	1990-1991	Spain	Female breast cancer	CC	7	F	18-75	762	988	O (5)	g/d (5)
Mattioli et al., 2006 [42]	NR	Italy	Atrial fibrillation	CC	6	M&F	54	116	116	E (3), I (3)	ml/d (4)
McFadden et al., 2009 [35]	1993-1997	UK	Stroke	CO	9	M&F	39-79	22,488	NA	O (5)	Units/wk (3)
Melchior et al., 2005 [28]	1989-1990	France	Cancers [Various]	CO	6	M&F	35-50	20,346	NA	O (3), OM	Glasses/d (4)
Menvielle, et al., 2004 [17]	1989-1991	France	Head & neck cancer	CC	7	M	NR	504	242	E (3), O (3)	Glasses/d (6)
Petrovski et al., 2011 [43]	2005	Hungary	Liver disease	CC	5	M	55 cases; 54 controls	287	892	E (4)	Units/wk (4); Problem drinking (2)
Radi et al., 2005 [41]	1997-1998	France	Hypertension	CC	5	M&F	42 M; 44 F	203	406	E (3)	Glasses/d (2)
Robert et al., 2004 [29]	1988-1995	USA	Female breast cancer	CC	7	F	62 cases; 61 controls	7,179	7,488	E (4), A (5)	Drinks/d (3)
Sharp et al., 2001 [21]	1993-1996	UK	Oesophageal cancer	CC	6	F	NR	159	159	O (5)	Units/wk (4)
Sundell et al., 2008 [36]	1987, 1992 & 1997	Finland	Stroke	CO	8	M&F	25-64	15,965	NA	E (3)	Binge drinking (2)
Veronesi et al., 2010 [37]	1986–1994	Italy	Stroke	CO	8	M&F	51	5,084	NA	E (2)	g/d (3)
Zatonski et al., 1991 [18]	1986-1987	Poland	Head & neck cancer	CC	7	M	53 cases; 44 controls	249	965	E (3), O (3)	Yrs vodka consumption (4)

NR, not reported. NA, not applicable. M, males. F, females. M&F, males and females. E, level of education. O, occupational social class. I, income. A, measure of area-level deprivation. ES, employment status. T, housing ownership or tenure. OM, occupational mobility. ml, millilitres. g, grams. yr, year. d, day. wk, week, BP, blood pressure.

**Table 2:** **Summary of quality assessment for cohort studies**

**Study, Year**	**Selection**				**Comparability**		**Outcome**			**QA**
	**1**	**2**	**3**	**4**	**5a**	**5b**	**6**	**7**	**8**	**Score**
Braaten et al., 2005 [22]	*	*	⋅⋅⋅	*	*	*	*	*	⋅⋅⋅	7
Brummett et al., 2011 [38]	*	*	⋅⋅⋅	⋅⋅⋅	*	*	⋅⋅⋅	*	⋅⋅⋅	5
Chaix et al., 2010 [39]	*	*	⋅⋅⋅	*	*	*	*	⋅⋅⋅	⋅⋅⋅	6
Dyer et al., 1999 [40]	*	*	*	*	*	*	*	*	*	8
Heck & Palmuck, 2007 [23]	*	*	*	*	*	*	*	*	⋅⋅⋅	8
Joshi et al., 2008 [30]	⋅⋅⋅	*	⋅⋅⋅	*	*	*	*	*	*	7
Kivimaki et al., 2009 [32]	*	*	⋅⋅⋅	*	*	*	*	*	⋅⋅⋅	7
Kuper et al., 2007 [33]	*	*	⋅⋅⋅	*	*	*	*	*	*	8
Laaksonen et al., 2007 [34]	*	*	⋅⋅⋅	⋅⋅⋅	*	*	*	*	*	7
Larsen et al., 2011 [26]	*	⋅⋅⋅	*	*	*	*	*	*	*	7
McFadden et al., 2009 [35]	*	*	*	*	*	*	*	*	⋅⋅⋅	9
Melchior et al., 2005 [28]	⋅⋅⋅	*	⋅⋅⋅	*	*	*	*	*	⋅⋅⋅	6
Sundell et al., 2008 [36]	*	*	⋅⋅⋅	*	*	*	*	*	*	8
Veronesi et al., 2010 [37]	*	*	*	*	*	*	*	*	⋅⋅⋅	8

*, criteria met. ⋅⋅⋅, criteria not met. Criteria: 1, Representative of average adult in the community. 2, Drawn from same community as exposed cohort. 3, Secure record or structured interview. 4, Demonstrated. 5a = Yes (age, sex, alcohol & SES). 5b = Yes (additional e.g. lifestyle factors). 6 = Independent blind assessment, record linkage. 7 = Follow-up > 6 months. 8 = Complete follow-up, or number lost <20%.

**Table 3:** **Summary of quality assessment for case–control studies**

**Study, Year**	**Selection**				**Comparability**		**Exposure**			**QA score**
	**1**	**2**	**3**	**4**	**5a**	**5b**	**6**	**7**	**8**	
Boing et al., 2011 [13]	⋅⋅⋅	⋅⋅⋅	⋅⋅⋅	⋅⋅⋅	*	*	⋅⋅⋅	*	*	5
Brown et al., 2001 [19]	*	*	⋅⋅⋅	⋅⋅⋅	*	*	*	*	*	7
Brown et al., 2005 [31]	⋅⋅⋅	*	*	*	*	*	⋅⋅⋅	*	⋅⋅⋅	6
Conway et al., 2010 [14]	*	*	*	⋅⋅⋅	*	*	⋅⋅⋅	*	*	7
Day et al., 1993 [15]	⋅⋅⋅	⋅⋅⋅	*	*	*	⋅⋅⋅	⋅⋅⋅	*	*	5
Gammon et al., 1997 [20]	*	*	*	*	⋅⋅⋅	⋅⋅⋅	⋅⋅⋅	*	*	6
Greenberg et al., 1991 [16]	⋅⋅⋅	⋅⋅⋅	*	*	*	⋅⋅⋅	⋅⋅⋅	*	*	5
Kropp et al., 2001 [24]	⋅⋅⋅	*	*	⋅⋅⋅	*	*	*	*	*	7
Kruk et al., 2007 [25]	⋅⋅⋅	*	⋅⋅⋅	*	*	*	*	*	*	7
Martin-Moreno et al., 1993 [27]	⋅⋅⋅	*	*	⋅⋅⋅	*	*	*	*	*	7
Mattioli et al., 2005 [42]	*	⋅⋅⋅	⋅⋅⋅	*	*	*	*	*	⋅⋅⋅	6
Menvielle et al., 2004 [17]	*	*	⋅⋅⋅	⋅⋅⋅	*	*	*	*	*	7
Petrovski et al., 2011 [43]	*	*	⋅⋅⋅	*	⋅⋅⋅	*	⋅⋅⋅	*	⋅⋅⋅	5
Radi et al., 2005 [41]	⋅⋅⋅	⋅⋅⋅	*	*	*	*	⋅⋅⋅	*	⋅⋅⋅	5
Robert et al., 2004 [29]	⋅⋅⋅	*	*	*	*	*	⋅⋅⋅	*	*	7
Sharp et al., 2001 [21]	*	*	*	⋅⋅⋅	⋅⋅⋅	*	*	*	⋅⋅⋅	6
Zatonski et al., 1991 [18]	*	*	*	⋅⋅⋅	*	*	⋅⋅⋅	*	*	7

*, criteria met. ⋅⋅⋅, criteria not met. 1, Yes, independent validation. 2, Consecutive or obviously representative series of cases. 3, Community controls. 4, No history of disease (endpoint). 5a, Yes (age, sex, alcohol & SES). 5b, Yes (additional e.g. lifestyle factors). 6, Secure record, or structured interview where blind to case/control status. 7, Yes, same method. 8, Same rate for both groups.

It was possible to explore the relationship between alcohol-attributable disease and SES across 26 studies [13-23,25-30,32-35,37,40-43]. These findings are summarised in Table 4, which presents the extracted unadjusted and adjusted risk estimates for low compared to high SES for each of these studies. The outcomes of the pooled analyses of this relationship are shown in Table 5 and discussed further below.

**Table 4:** **Relationship between alcohol-attributable disease and SES**

**Study**	**SES measure**	**Sex**	**RR/OR/HR (95% CI) for low compared to high SES**		**Variables adjusted for**
			**Unadjusted**	**Adjusted**	
*Head & neck cancer*					
Boing et al., 2011 [13]	Education	M + F	2.27 (1.61, 3.19)	1.58 (1.06, 2.36)	Age, sex, smoking, alcohol use
Conway et al., 2010 [14]	Neighbourhood^a^	M + F	3.62 (1.35, 9.71)	1.90 (0.59, 6.09)	Age, sex, smoking, alcohol use
Day et al., 1993 [15]	Education	M + F	· · ·	White: 1.40 (1.00, 1.80)	Smoking, alcohol use
				Black: 1.20 (0.60, 2.60)	
Greenberg et al., 1991 [16]	Education	M	· · ·	1.0 (0.7, 1.5)	Age, ethnicity, marital status, study area, smoking, alcohol use, snuff dipping, tobacco chewing, tooth loss, denture problems, education, occupational status, percentage of years worked
Menvielle, et al., 2004 [17]	Education	M	3.22 (2.01, 5.18)	1.63 (0.90, 2.98)	Age, smoking, alcohol use
Zatonski et al., 1991 [18]	Education	M	2.94 (2.03, 4.27)	2.51 (1.06, 5.94)	Age, smoking, alcohol use
*Female breast cancer*					
Braaten et al., 2005 [22]	Education	F	0.68 (0.56, 0.83)	0.90 (0.73, 1.12)	Parity, alcohol use, OC, height, HRT, BMI, mammography, menopausal status
Heck & Pamuk, 1997 [23]	Education	F	0.44 (0.24, 0.80)	0.66 (0.37, 1.19)	Age, education, income, ethnicity, family history of breast cancer, parity, age at menarche, age at menopause, OC, HRT, alcohol use, BMI, height
Kruk et al., 2007 [25]	Education	F	· · ·	Pre-menopausal: 2.39 (1.58, 3.60)	Age, BMI, stress experience, passive smoking
				Post-menopausal: 1.31 (0.98, 1.76)	
Larsen et al., 2011 [26]	Education	F	0.84 (0.70, 1.00)	0.94 (0.79, 1.12)	HRT, parity*,* alcohol use, BMI
Martin-Moreno et al., 1993 [27]	OSC	F	· · ·	0.63 (0.33, 1.17)	Age, study area, SES, BMI, total energy intake
Melchior et al., 2005 [28]	OSC	F	0.92 (0.44, 1.92)	0.96 (0.46, 2.03)	Age, smoking, alcohol use, marital status, BMI, FVC, family history of breast cancer, age at first childbirth
Robert et al., 2004 [29]	Education	F	0.87 (0.77, 0.98)	0.96 (0.84, 1.10)	Age, interview year, community SES, urbanicity, mammography, family history of breast cancer, parity, alcohol use, BMI, age at first birth, HRT, OC
*Oesophageal cancer*					
Brown et al., 2001 [19]	Education	M	· · ·	White: 1.50 (0.90, 2.60)	Age, study area, alcohol use, smoking, FVC
		M	· · ·	Black: 3.10 (1.60, 6.10)	
Gammon et al., 1997 [20]	Income	M	· · ·	5.00 (1.67, 14.99)	Age, sex, study area, ethnicity, BMI, smoking, alcohol use
Sharp et al., 2001 [21]	OSC	F	1.51 (0.75, 3.06)	· · ·	Not applicable
*Other cancers*					
Braaten et al., 2005 [22] *All cancers*	Education	F	0.95 (0.85, 1.07)	· · ·	Not applicable
Melchior et al., 2005 [28] *All cancers*	OSC	M	1.47 (1.03, 2.09)	1.35 (0.94, 1.93)	Age, smoking, alcohol use, marital status, BMI, FVC, family history of lung & oral cancer (M), occupational asbestos exposure (M), family history of breast cancer (F), parity (F)
		F	1.00 (0.58, 1.73)	1.03 (0.59, 1.80)	
Braaten et al., 2005 [22] *Colon cancer*	Education	F	1.23 (0.70, 2.18)	· · ·	Not applicable
Braaten et al., 2005 [22] *Rectal cancer*	Education	F	0.63 (0.33, 1.21)	· · ·	Not applicable
Joshi et al., 2008 [30] *Liver cancer*	OSC	M	1.75 (1.48, 2.07)	1.63 (1.38, 1.93)	Age, fasting serum glucose, BMI, alcohol use, smoking
Melchior et al., 2005 [28] *Smoking/alcohol-related cancers* ^*b*^	OSC	M	2.18 (1.15, 4.11)	1.54 (0.80, 2.97)	Age, smoking, alcohol use, marital status, BMI, FVC, family history of lung & oral cancer, occupational asbestos exposure
*Stroke*					
Kivimaki et al., 2009 [32]	OSC	F	2.28 (1.30, 3.90)	1.88 (1.10, 3.20)	Hypertension, CHD, diabetes, smoking, alcohol use, PA, BMI
Kuper et al., 2007 [33]	Education	F	2.10 (1.40, 2.90)	1.80 (1.30, 2.60)	Age, alcohol use
Laaksonen, et al., 2008 [34]	Education	M + F	1.59 (0.84, 2.99)	1.57 (0.83, 2.96)	Age, age squared, study year, diabetes, myocardial infarction, CHD, heart failure, alcohol use
McFadden et al., 2009 [35]	OSC	M	2.84 (1.40, 5.74)	2.90 (1.43, 5.87)	Age, alcohol use
		F	2.32 (1.19, 4.49)	2.05 (1.05, 4.00)	
Veronesi et al., 2010 [37]	Education	M	2.14 (1.25, 3.69)	2.18 (1.26, 3.78)	Age, hypertension, diabetes, smoking, HDL cholesterol, alcohol use
		F	0.54 (0.26, 1.12)	0.40 (0.20, 0.85)	
*Hypertension*					
Dyer et al., 1999 [40]	Education	M	Black: 0.98 (0.72, 1.31)	Black: 1.01 (0.72, 1.41)	Age, systolic BP, BMI, waist circumference, PA, alcohol use, pulse, smoking, education, fasting insulin, triglycerides, uric acid and HDL cholesterol
			White: 0.57 (0.37, 0.86)	White: 0.48 (0.30, 0.76)	
		F	Black: 0.65 (0.47, 0.89)	Black: 0.67 (0.48, 0.94)	
			White: 0.25 (0.15, 0.42)	White: 0.42 (0.23, 0.78)	
Radi et al., 2005 [41]	OSC	F	· · ·	8.12 (1.30, 50.73)	Age, education, smoking (women), alcohol use, PA, social support at work, recent stressful life events, low support outside of work (women)
*Other conditions*					
Mattioli et al., 2006 [42] *Atrial fibrillation*	Education	M + F	1.12 (0.50, 2.49)	· · ·	Not applicable
Petrovski et al., 2011 [43] *Liver disease*	Education	M + F	3.22 (1.72, 6.03)	2.86 (1.30, 6.26)	Age, smoking, alcohol use, PA

^a^Scottish Index of Multiple Deprivation score. ^b^Cancers of the oral cavity and pharynx, oesophagus, pancreas, larynx, trachea & lung, urinary tract. OR, odds ratio. RR, relative risk. HR, hazard ratio. CI, confidence interval. M + F, sex data combined. OSC, occupational social class. · · ·, not reported. BMI, body mass index. CHD, coronary heart disease. FVC, fruit & vegetable consumption. HR, heart rate. HRT, hormone replacement therapy. OC, oral contraceptives. PA, physical activity SES, socioeconomic status.

**Table 5:** **Meta-analysis results: random effects pooled risk estimates for low compared to high SES groups**

**Disease/Condition**	**Studies**	**Pooled risk estimate**	**I** ^**2**^ **statistic**
*Unadjusted*			
Head and neck cancer	4	2.72 (2.20, 3.37)	0%
Female breast cancer	5	0.78 [0.67, 0.91]	53%
Stroke	5	1.84 [1.33, 2.56]	59%
*Adjusted*			
Head and neck cancer	6	1.40 [1.18, 1.66]	0%
Female breast cancer	7	1.04 [0.85, 1.27]	73%
Stroke	5	1.65 [1.13, 2.41]	67%

We used the *I* ^*2*^ statistic (95% CI) to estimate heterogeneity between pooled studies: *I*2 = 30–60%, moderate heterogeneity; 50–90%, substantial heterogeneity; 75–100%, considerable heterogeneity.

### Alcohol-attributable cancers and SES

**Figure 2:**
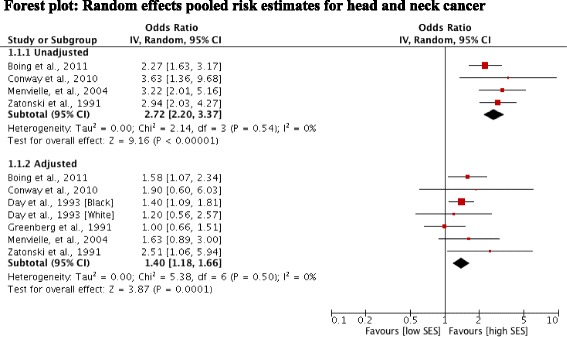
Forest plot: Random effects pooled risk estimates for head and neck cancer.

Four studies [13,14,17,18] reported unadjusted outcomes for cancers of the head and neck. Pooling of these estimates revealed a statistically significant positive association between low SES and cancers of the head and neck (OR 2.72; 95% CI 2.20, 3.37). Heterogeneity was low indicating the consistency of an increased risk among low SES groups in these studies (Figure 2). Adjustment for smoking and alcohol use was examined jointly in these studies. Pooling data across seven studies [13-18] of head and neck cancer risk showed that significance was retained after adjustment (OR 1.40; 95% CI 1.18, 1.66). Again a low level of heterogeneity indicated consistency of the findings. In general, studies found that differences in alcohol and smoking behaviours between SES groups did not fully explain the relationship between low SES and head and neck cancer. Menvielle et al. [17] noted that a substantial proportion of the risk in their study was explained by occupational exposures, but these factors were not fully considered in the other studies.

**Figure 3:**
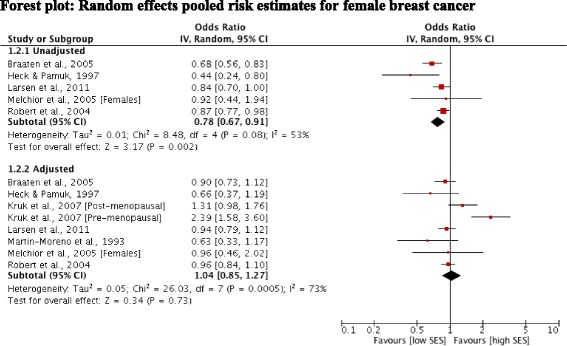
Forest plot: Random effects pooled risk estimates for female breast cancer.

Five studies [22,23,26,28,29] reported outcomes for female breast cancer. Pooling of the unadjusted findings for these five studies showed a significant positive association between risk of breast cancer and high SES (OR 0.78; 95% CI 0.67, 0.91). However a high level of heterogeneity indicated inconsistency across the study estimates (Figure 3). Seven studies [22,23,25-29] reported odds ratios adjusted for risk factors for female breast cancer (including factors such as parity, use of HRT and BMI alongside alcohol use). Pooling of the adjusted estimates changed the direction of the effect (OR 1.04; 95% CI 0.85, 1.27). However, statistical tests again revealed a high level of heterogeneity and visual inspection identified the estimates from the study by Kruk et al. [25] as outliers. After exclusion of this study, heterogeneity was substantially reduced and revealed a positive, but non-significant association between risk of breast cancer and high SES (OR 0.93; 95% CI 0.84, 1.02).

Oesophageal cancer risk was explored in three studies [19-21]. Pooling was not feasible as adjusted risk estimates were only reported for two studies. Individually these studies showed mixed findings; after adjustment for a range of lifestyle factors (including alcohol use and smoking), two studies [19,20] found a statistically significant association between oesophageal cancer risk for income but not occupational social class, or level of education (data not shown). Three studies [22,28,30] examined other types of cancer but meta-analysis was not feasible as insufficient data were available. Two studies reported statistically significant associations between risk of liver cancer and low social class [30], all cancers and low social class among men and smoking and alcohol-related cancers and low social class among men [28]. After adjustment for lifestyle factors including alcohol use, Melchior et al. [28] reported that observed gradients in risk of all cancers and smoking and alcohol-related cancers were non-significant. Joshi et al. [30] found that adjusting for alcohol use and other factors did not attenuate a significant association between SES and liver cancer risk.

### Alcohol-attributable cardiovascular disease and SES

**Figure 4:**
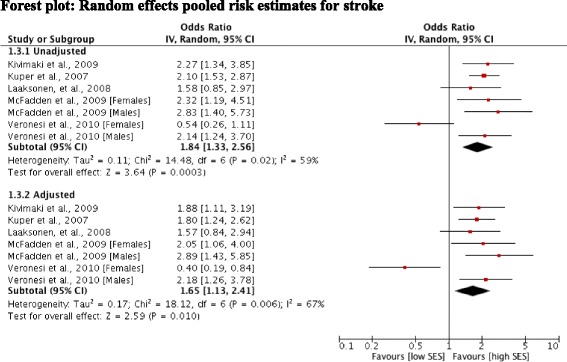
Forest plot: Random effects pooled risk estimates for stroke.

Six studies [31-35,37] reported outcomes for stroke risk. One study [31] reported regression coefficients and was not included in the meta-analysis. Pooled unadjusted data from five studies showed a positive, statistically significant relationship between low SES and stroke risk (OR 1.84; 95% CI 1.33, 2.56). The remaining studies all reported odds ratios after adjustment for alcohol use and other conventional risk factors for stroke. Adjustment attenuated the excess risk but the pooled estimate showed that low SES remained significantly associated with risk of death or hospitalisation from stroke (OR 1.65; 95% CI 1.13, 2.41) (Figure 4). In these five studies [31-33,35,37] assessment of heterogeneity indicated inconsistency between the adjusted and unadjusted study estimates (I^2^ = 67% and 59%, respectively). Excluding the study by Veronesi et al. [37] substantially reduced the heterogeneity across both the unadjusted and adjusted pooled estimates; giving ORs of 2.14 (95% CI: 1.71, 2.66) for the unadjusted estimates and 1.91 (95% CI: 1.51, 2.43) for the adjusted estimates. In this study, separate estimates were presented for men and women, with the direction of the association with level of education reversed for women (i.e. lower risk associated with a low level of education) but not men. Brown et al. [31] found that lower SES was associated with a higher risk of stroke.

Two studies [40,41] reported a statistically significant association between risk of hypertension and a low level of education and low level of social class, respectively. Both studies adjusted for common risk factors for hypertension (including alcohol use) and individually, for measures associated with job constraints [41] and metabolic syndrome X variables [40]. Two studies [38,39] reported the outcomes of an association between systolic blood pressure and SES as regression coefficients (data not shown). Both studies found an association between a low level of education and increases in systolic blood pressure, but after adjustment for behavioural and lifestyle factors such as alcohol use, an association remained only in the study by Chaix et al. [39].

### Other alcohol-attributable conditions and SES

In unadjusted models, risk of liver disease was associated with a low level of education [43], whereas atrial fibrillation had no association with education or income [42]. After adjustment for behavioural factors including alcohol use, risk of liver disease remained significantly associated with a low level of education [43].

### Relationship between alcohol-attributable disease, SES and alcohol use

The majority of studies included alcohol use as a potential confounder alongside other risk factors (for example, smoking, fruit and vegetable consumption, BMI) and it was therefore only possible to examine the relationship between SES and alcohol use alone in two studies of stroke risk [33,35]. In addition, four studies [13,14,17,18] of cancers of the head and neck reported models adjusted for alcohol use in combination with smoking.

As a mediator of the socioeconomic gradient in stroke risk, Kuper et al. [33] found that adjustment for alcohol use explained 27% and 16% of the difference in risk between high and low education groups for all strokes and ischaemic strokes, respectively. McFadden et al. [35] stratified their analyses by sex, finding that alcohol use accounted for 21% of the difference between high and low income groups among women but did not substantially decrease the odds ratio among men (<5% difference). Low SES remained an independent predictor of stroke in adjusted models in both studies. For cancers of the head and neck, including smoking and alcohol use as covariates accounted for between 9% [18] and 72% [17] of the difference in risk between high and low education groups, and between 41% [17] and 61% [18] of the difference in risk between high and low income groups. In one study [14], smoking and alcohol use in combination accounted for 66% and 71% of the difference in risk between high and low SES groups according to two separate measures of area-level deprivation. Two studies examined the role played by alcohol use as a possible mediating factor in the relationship between education and systolic blood pressure [38,39]. Alcohol use increased with education in both studies with Chaix et al. [39] noting that this finding “tended to mask rather than explain” the association between education and systolic blood pressure.

Two studies [19,24] reported SES measures stratified by alcohol consumption. Brown et al. [19] examined the combined effects of alcohol use and smoking, finding that while increasing risks were seen for each income and alcohol use category, risks were highest among heavy drinkers in the lowest income category. Kropp et al. [24] found that education status modified the effect of alcohol use on the risk of breast cancer. Risks were significantly increased among women in the highest alcohol consumption category who reported a low or intermediate level of education, compared with no significant risk among women with a high level of education. The risk estimates describing these relationships are presented in Table 6.

**Table 6:** **Stratification of SES measures by alcohol consumption categories**

**Author, Year**	**Disease/condition**	**Alcohol measure**	**SES measure**	**SES level**	**RR/OR/HR (95% CI)**	**Variables adjusted for**
Brown et al., 2001 [19]	Oesophageal cancer	15–35 drinks/wk^a^	Income	Low	71.80 (15.00, 343.90)	Age, study area, raw fruit and vegetable consumption, ethnicity
				Intermediate	14.60 (2.90, 73.80)	
				High	2.00 (0.20, 23.10)	
		>35 drinks/wk^a^	Income	Low	231.60 (48.20, 1114.00)	
				Intermediate	98.80 (20.90, 467.30)	
				High	38.70 (7.10, 210.40)	
Kropp et al., 2001 [24]	Breast cancer	≥19 g/d	Education	Low	3.70 (1.23, 11.15)	Parity, breastfeeding, education, menopausal status, family history of breast cancer
				Intermediate	1.57 (1.03, 2.35)	
				High	0.70 (0.39, 1.27)	

^a^Data presented for light smokers category only.

## Discussion

Due to limitations in the data identified, the aims of this systematic review were repurposed to examine the role of alcohol consumption in the relationship between risk of alcohol-attributable disease and socioeconomic indicators, primarily level of education. The included studies covered a range of alcohol-attributable conditions, and we identified differing relationships between the selected conditions and socioeconomic indicators. Our pooled analyses showed that low, relative to high SES, was associated with an increased risk of head and neck cancer and stroke, and in individual studies, with hypertension and liver disease. Conversely, risk of female breast cancer tended to be associated with higher SES. These findings also held in models adjusted for a number of known risk factors and other potential confounding factors.

A key finding of our review is the lack of studies that have explored in depth, the relationship between alcohol-attributable disease, socioeconomic status and alcohol use. In studies that adjusted for alcohol use independent of other lifestyle risk factors, its addition to statistical models explained a substantial proportion of the difference in risk between high and low SES groups for stroke risk, and in combination with smoking, head and neck cancer risk. Interaction was explored in two studies, of female breast cancer and oesophageal cancer risk, respectively. These studies showed that when SES measures were stratified by alcohol use, risks were greatest among low SES groups. Therefore, it may be that for some conditions, and demonstrated here for female breast cancer and hypertension, that alcohol consumption has a tendency to mask rather than explain the associations between SES and disease risk.

One of the main limitations of the review was the lack of sufficient data to conduct a meta-analysis of risk estimates stratified by SES and, therefore, our inability to fully explore the relationship between alcohol use and SES in the risk of alcohol-attributable disease. We have been unable as a consequence to estimate the overall magnitude of the association between SES, alcohol consumption, and alcohol-attributable disease risk. We are confident that our methodology was robust and comprehensive. We undertook a thorough search of the literature and retrieved a high volume of references. However, we should acknowledge the following limitations of the search; due to resource constraints we excluded articles with languages other than English and did not incorporate a search of the grey literature. Furthermore, our inclusion criteria were limited to case–control and cohort designs, and to studies of participants older than 16 years. So while in practice we found that relatively few studies presented a sufficient level of information to enable a joint analysis of SES and alcohol consumption, we acknowledge that the limitations of our search strategy and inclusion criteria may in part have contributed to the lack of study identification. It is unclear whether the lack of evidence on the interaction between SES and alcohol consumption implies that there is evidence of a lack of significant interactions for the conditions examined. Future work in this area may prove more successful if different methodological approaches are adopted, such as individual patient data meta-analysis or secondary analysis of cohorts. Further limitations in our approach may have arisen through the use of different definitions and measures of SES and alcohol consumption across the included studies, and through the presence of confounding bias. Different risk factors were adjusted for in the included studies, including by disease area, and we should therefore assume that residual confounding persists across the included studies. Our broad inclusion criteria for conditions are likely to have meant that the search strategy lacked specificity, reflected in the large volume of references retrieved. As noted, future work may benefit from taking a condition by condition approach.

## Conclusions

Whilst acknowledging the scarcity of the evidence available, the findings of this review does provide further evidence that people of low SES show a greater susceptibility to the damaging effects of alcohol [44]. However, the mechanisms and pathways underlying this differential risk remain unclear and require further study. Explanatory mechanisms that have been proposed for the association between risk of alcohol-attributable disease and SES include the direct effects of: (i) differences in drinking behaviours, including quality of the alcohol consumed [45-52]; (ii) interaction through clustering of risky lifestyle behaviours, such as heavy alcohol use and smoking [53]; and (iii) differential access to healthcare [54]. Other hypothesised mechanisms include differences in the availability of social support [44] and drinking context, such as where and with whom drinking occurs [55]. Neighbourhood deprivation, acting both independently of, and in interaction with, individual SES is also thought to play a role [56,57].

Despite the limitations of our review, we have described relationships between SES and a range of alcohol-attributable conditions, and explored the mediating effects of alcohol consumption where feasible. However, further research is needed to better characterise the interaction between SES, alcohol consumption and alcohol-attributable disease risk so as to gain a greater understanding of the mechanisms and pathways that influence the potentially differential risk.

## Additional file

Additional file 1:
**Search Strategy for MEDLINE via OVID.**
