# Supplementary material for: Relationship between alcohol-attributable disease and socioeconomic status, and the role of alcohol consumption in this relationship: a systematic review and meta-analysis
Source: BMC Public Health. 2015 Apr 18;15:400. doi: 10.1186/s12889-015-1720-7 (PMC4409704; doi:10.1186/s12889-015-1720-7)
Supplement: Additional file 1: — Search Strategy for MEDLINE via OVID. [file 12889_2015_1720_MOESM1_ESM.docx]

**Additional file 1 Search Strategy for MEDLINE via OVID**

| **#** | **Terms** |
| --- | --- |
| 1 | Socioeconomic factors/ |
| 2 | exp Social class/ |
| 3 | Social conditions/ |
| 4 | exp Poverty/ |
| 5 | Marital status/ |
| 6 | Career mobility/ |
| 7 | Psychosocial deprivation/ |
| 8 | Maternal deprivation/ |
| 9 | Paternal deprivation/ |
| 10 | Occupations/ |
| 11 | Educational status/ |
| 12 | exp Income/ |
| 13 | Employment/ |
| 14 | (socio?economic OR SES OR SEP OR socio?demographic OR poverty OR deprived OR deprivation OR disadvantage* OR income OR earning* OR marital status).ti,ab. |
| 15 | (social ADJ1 (class* OR group* OR factor* OR condition* OR circumstance* OR mobil*)).ti,ab. |
| 16 | (employment status OR occupation* OR manual OR non-manual).ti,ab. |
| 17 | (education* ADJ (level OR attain*)).ti,ab. |
| 18 | or/1-17 |
| 19 | *Alcohol drinking/ |
| 20 | exp Alcoholic beverages/ |
| 21 | Alcohol-related disorders/ |
| 22 | Alcoholism/ |
| 23 | Alcoholic intoxication/ |
| 24 | (alcohol* ADJ (drink OR drinks OR beverage*)).ti,ab. |
| 25 | ((alcohol OR ethanol) adj1 (consumption OR drinking OR intake OR abuse OR misuse)).ti,ab. |
| 26 | ((harmful OR hazardous OR problem OR risky OR heavy OR excessive OR binge OR light OR moderate) adj1 drinking).ti,ab. |
| 27 | (drinking behavio?r OR beer OR wine OR spirits OR absinthe OR liquor*).ti,ab. |
| 28 | or/19-27 |
| 29 | exp Alcohol Induced Disorders/ |
| 30 | (alcohol* ADJ (polyneuropathy OR myopathy OR cardiomyopathy OR gastritis OR liver disease OR steatosis OR fibrosis OR hepatitis OR cirrhosis OR pancreatitis OR psychoses)).ti,ab. |
| 31 | exp Mouth Neoplasms/ |
| 32 | Esophageal Neoplasms/ |
| 33 | Colorectal Neoplasms/ |
| 34 | exp Liver Neoplasms/ |
| 35 | exp Breast Neoplasms/ |
| 36 | Laryngeal Neoplasms/ |
| 37 | ((oesophageal OR esophageal OR colorectal OR colon OR rectal OR liver OR breast OR larynx OR laryngeal OR lip OR oral cavity OR pharynx OR pharyngeal OR tongue OR gum OR mouth OR palate OR parotid gland OR salivary gland* OR tonsil OR oropharynx OR nasopharynx OR piriform sinus OR hypopharynx) ADJ (neoplasm* OR cancer*)).ti,ab. |
| 38 | exp Epilepsy/ |
| 39 | (epilep* OR seizures).ti,ab. |
| 40 | exp Hypertension/ |
| 41 | (hypertension OR hypertensive).ti,ab. |
| 42 | exp Arrhythmias, Cardiac/ |
| 43 | (rhythm* OR arrhythmia OR dysrhythmia OR tachyarrhythmia OR bradyarrhythmia OR tachycardia OR bradycardia OR conduction OR atrial flutter OR ventricular flutter OR atrial fibrillation OR ventricular fibrillation OR paroxysmal OR exstrasystol*).ti,ab. |
| 44 | exp Cerebrovascular Disorders/ |
| 45 | (stroke* OR cerebrovascular* OR (intracranial ADJ (embolism OR thrombosis))).ti,ab. |
| 46 | Liver diseases/ |
| 47 | Liver cirrhosis/ |
| 48 | Fatty liver/ |
| 49 | (liver disease* OR steatosis OR fibrosis OR hepatitis OR cirrhosis).ti,ab. |
| 50 | exp Pancreatitis/ |
| 51 | (pancreatitis).ti,ab. |
| 52 | or/29-51 |
| 53 | exp "Wounds and Injuries"/ |
| 54 | exp Accidents/ |
| 55 | exp Poisoning/ |
| 56 | exp Suicide/ |
| 57 | exp Self Injurious Behavior/ |
| 58 | (poison* OR injur* OR trauma* OR fall OR falls OR burn* OR fire* OR flame* OR drown* OR scald* OR crash* OR accident* OR suicid* OR assault* OR murder* OR homicid* OR attack* OR stab OR stabbed OR stabbing* OR self harm* OR self-harm* or self-injur*).ti,ab. |
| 59 | or/53-58 |
| 60 | exp Case-Control Studies/ OR exp Cohort studies/ OR Control groups/ |
| 61 | (case OR cohort OR control group* OR ratio OR risk* OR prospective* OR follow* OR longitudinal OR retrospective).ti,ab. |
| 62 | 60 OR 61 |
| 63 | 18 AND 28 AND 52 AND 62 |
| 64 | 18 AND 28 AND 59 AND 62 |
| 65 | Animals/ NOT Humans/ |
| 66 | 63 NOT 65 |
| 67 | limit 66 to yr=1990 - current |
| 68 | 64 NOT 65 |
| 69 | limit 68 to yr=1990 - current |
| 70 | 67 or 69 |
